# Mycotoxins and Beyond: Unveiling Multiple Organic Contaminants in Pet Feeds Through HRMS Suspect Screening

**DOI:** 10.3390/toxins18010022

**Published:** 2025-12-30

**Authors:** Dafni Dematati, Dimitrios Gkountouras, Vasiliki Boti, Triantafyllos Albanis

**Affiliations:** 1 Department of Chemistry, University of Ioannina, GR-45110 Ioannina, Greece; dafnidematati@gmail.com (D.D.); d.gkountouras@uoi.gr (D.G.); 2 Unit of Environmental, Organic and Biochemical High-Resolution Analysis–Orbitrap-LC–MS, University of Ioannina, GR-45110 Ioannina, Greece

**Keywords:** animal feeds, suspect screening, LC-HRMS, feed safety, pet feed contamination, mycotoxins, pesticides, veterinary drugs

## Abstract

This study evaluated 65 commercially available pet feed samples, including 33 cat feeds and 32 dog feeds (dry and wet formulations), for the presence of organic contaminants. These included mycotoxins, pesticides, pharmaceutical residues/veterinary drugs, and plant-based bioactive compounds. A suspect screening strategy was employed using QuEChERS extraction followed by LC-LTQ/Orbitrap HRMS analysis. A total of 29 compounds were tentatively identified within 186 detections. In total, 76.9% of the samples were contaminated with mycotoxins. Aflatoxins (B1, B2, G1, and G2), T2 toxins, and HT2 toxins were dominant, with Aflatoxin B1 occurring in 33.8% of the samples and exhibiting a higher prevalence in dry feeds than in wet feeds. Pesticides were present in 72.0% of the dry formulations, including aclonifen and pirimiphos-methyl, but were present in only 11% of the wet formulations. Plant-based bioactive compounds, including phytoestrogens, were identified in 51% of the samples, highlighting toxicologically relevant candidates that merit prioritization for targeted confirmation, particularly in cat feeds. Pharmaceuticals were found in 23.8% of dry feeds (sparfloxacin and fumagillin). Overall, the HRMS-based, standard-free suspect screening workflow provides an early-warning overview of multi-class co-occurrence patterns in complex pet feed matrices and supports the prioritization of candidates for subsequent confirmatory analysis.

## 1. Introduction

Organic contaminants, including mycotoxins, pesticides, pharmaceuticals/veterinary drugs, and certain plant-based bioactive compounds, pose significant risks to animal and human health [1,2]. These compounds may enter the food chain through multiple routes, including crop contamination, environmental exposure, and the use of low-quality raw materials in feed production [3]. Even at low concentrations, mycotoxins can induce toxicogenic reactions in higher vertebrates and other animals when introduced via direct or indirect exposure [4,5,6].

Among these, mycotoxins, which are naturally occurring secondary metabolites produced by several fungal species such as *Aspergillus*, *Penicillium*, and *Fusarium*, have received particular attention because of their persistence and toxicity [4,7,8,9]. These compounds have been implicated in both acute and chronic health disorders in humans and animals owing to their chemical properties. Furthermore, toxic transformation products (TPs) can survive food processing steps, leading to contamination of final food products [10,11] and pose significant health risks, including immune suppression, carcinogenicity, pathological lesions, and growth impairments [12,13,14]. While livestock feed undergoes regulatory monitoring, systematic monitoring of commercial pet feed for multi-class contaminants remains fragmented and underfunded. The proposed approach is useful for feed safety as an early-warning tool. It helps prioritize contaminants for follow-up by revealing co-occurrence patterns in complex feeds.

Animal feed, whether intended for pets or production species, incorporates a diverse array of raw materials such as grains, fruits, and proteins [1,15]. This complexity increases the risk of contamination by various classes of harmful compounds, including mycotoxins, pesticides, antibiotics, and natural metabolites with hormonal activity [1,16]. In particular, the vulnerability of feed ingredients to fungal contamination under improper moisture conditions presents a critical challenge to feed safety. Food crops and feed ingredients are particularly susceptible to mold and fungal growth when exposed to moisture, which is necessary for fungal proliferation [12,17]. Mycotoxin production by fungi can occur at multiple stages of the food production process, including preharvest, harvesting, drying, and storage [7,17]. Moisture levels above 14–16% are generally considered critical thresholds for mold proliferation in stored feed commodities [18]. In addition to fungal contamination, additional contaminant sources include pesticide residues from treated crops, veterinary pharmaceuticals present in animal-derived ingredients, and bioactive plant metabolites introduced through legumes or grains [1,4,9,19,20]. These compounds, while less commonly regulated than mycotoxins, may lead to chronic exposure and pose an extra safety risk for companion animals.

Owing to the significant impact of mycotoxins, several international bodies, including the European Commission (EU) [21], United States Food and Drug Administration (FDA) [22], and World Health Organization (WHO) [23], have established regulatory thresholds for permissible levels of mycotoxins in food and feed. For example, the limits for aflatoxin B1 in pet feed are specified in Annex I of Directive 2002/32/EC [24]. Unlike livestock, there is no specific separate category for complete pet feed in most cases, meaning pet food generally complies with the standard complete feed limits (feed materials, 0.02 mg/kg). Notably, Commission Recommendation 2013/637/EU [25] specifically addresses T-2 and HT-2 toxins in compound feed for cats, setting a guidance value of 0.05 mg/kg (50 μg/kg). The European Food Safety Authority (EFSA) and the Rapid Alert System for Food and Feed (RASFF) regularly monitor food and feed contamination across Europe and evaluate the potential risks that could threaten consumer health [4,7,15,26,27,28]. Despite decades of regulation, the occurrence of mycotoxins in crops and throughout the food production chain remains a persistent food safety challenge, particularly owing to the frequent co-occurrence of multiple mycotoxins in single commodities [26]. For pesticide residues, Regulation (EC) No 396/2005 [29] applies to livestock feed, whereas the specific category feed for non-food-producing animals remains empty without specific pesticide MRLs.

Although mycotoxins in food are routinely monitored, information regarding their occurrence in animal feed remains limited [10,30]. Most of the available studies have been performed on feed aimed at livestock production [31,32,33], whereas few studies have reported the occurrence of these toxic compounds in pet feeds [5,10,17,34,35]. Even fewer investigations have comprehensively addressed the simultaneous presence of pesticides, pharmaceuticals, and plant secondary metabolites in pet diets, despite their potential toxicological relevance. Given these considerations, multi-class HRMS suspect screening is particularly valuable as a standard-free, qualitative early-warning tool that maps co-occurrence patterns and supports the prioritization of contaminants for follow-up confirmatory and quantitative investigations.

Concerning the identification of organic contaminants, including mycotoxins and the transformation products (TPs), mass spectrometry techniques play the main role in advancing multiresidue analysis [10]. High-resolution mass spectrometry (HRMS), particularly when combined with suspect screening workflows, enables the detection of both targeted and non-targeted contaminants, even in the absence of analytical standards, thereby offering broader chemical coverage [36,37]. Moreover, HRMS, and particularly orbitrap-based systems, have become increasingly important in food and feed safety research. HRMS analyzers exhibit exceptional specificity and high resolution, which is attributed to the high mass accuracy and structural elucidation capabilities provided by MS/MS acquisition [1,10,38,39,40,41]. Along with instrumental advancements, the role of extraction methodologies remains critical because the efficient isolation of analytes is fundamental. Generic extraction techniques such as QuEChERS have gained substantial acceptance because of their efficiency, simplicity, and minimal sample preparation requirements [1,8,11,40,42,43].

In this study, an LC-HRMS suspect screening workflow was applied to 65 commercially available pet feeds (dog and cat) to identify emerging mycotoxins and characterize the co-occurrence of a plethora of contaminant classes, namely pesticide residues, pharmaceuticals/veterinary drugs and plant-derived bioactives. Previous pet feed studies have primarily focused on mycotoxins using HRMS, with limited chemical scope beyond fungal metabolites and without systematic multiclass screening in the same run [44]. By contrast, this study integrates multi-class suspect screening within a single analytical framework and compares contamination patterns across wet and dry formulations. Related multi-class HRMS approaches have been applied to individual matrices—mycotoxins in pet feed [10], various contaminants in aquafeeds [1,45] and pesticides/mycotoxins/plant toxins in food [46,47]—comprehensive, simultaneous mapping of all four contaminant classes in pet feeds remains undocumented. Therefore, the proposed approach expands chemical coverage in the context of pet nutrition and provides qualitative occurrence signals (tentative identifications) that enable early-warning interpretation and prioritization across formulation types (wet vs. dry).

## 2. Results and Discussion

### 2.1. Comparative Evaluation of Clean-Up Alternatives for Suspect Screening

Matrix effects in pet feed analysis can profoundly influence chromatographic performance and mass spectrometric detection, particularly in suspect screening techniques that simultaneously investigate compounds with diverse physicochemical properties and ionization behaviors. Thus, the optimal clean-up was selected before the primary analysis to reduce interference and enhance the overall reproducibility of the procedure. By comparing the performance of traditional sorbent mixtures with a newer alternative, the method development aimed to establish the most suitable cleanup approach for challenging matrices of dry and wet pet feeds.

This study assessed three dispersive solid-phase extraction (dSPE) clean-up combinations, encompassing both conventional and alternative methodologies. The evaluated protocols included (a) 150 mg MgSO_4_, 50 mg PSA, and 50 mg C18; (b) 300 mg MgSO_4_, 50 mg PSA, and 50 mg C18; and (c) 50 mg Z-Sep+. The studied combinations were used to investigate the impact of the amount of salt and sorbent on the quality of the extracts.

The evaluation of these clean-up alternatives was guided by their anticipated influence on chromatographic clarity, mitigation of matrix-related interference, and reliability of analyte identification. The three clean-up combinations were evaluated for six pet feeds; three dry formulations (6–15% moisture content) and three wet formulations (78–85% moisture content). The efficiency of each combination was evaluated based on the total amount and diversity of compounds reported through suspect screening. The cleanup of wet feeds using 300 mg MgSO_4_, 50 mg PSA, and 50 mg C18 (protocol b) provided the best results, with a total score of 17 identifications, thereby enabling the detection of analytes in all categories, including mycotoxins, pesticides, plant metabolites, and pharmaceuticals. On the other hand, the variation with diminished MgSO_4_ (protocol a) achieved a score of 6, mainly limited to a small number of mycotoxins and plant secondary metabolites, while Z-Sep+ (protocol c) demonstrated poor efficacy (score = 3), identifying only sporadic signals from distinct compound classes.

As far as the dry feeds are concerned, the optimal performance was achieved with a mixture of 150 mg MgSO_4_, 50 mg PSA, and 50 mg C18 (protocol a), resulting in the highest score of 23 identifications, with extensive coverage of mycotoxins, pesticides, plant metabolites, and pharmaceuticals. The use of 300 mg MgSO_4_ (protocol b) yielded a diminished score (17), hence decreasing the diversity of identified compounds, whereas Z-Sep+ (protocol c) proved to be inadequate (score = 5), predominantly linked to a limited number of mycotoxins.

**Figure 1:**
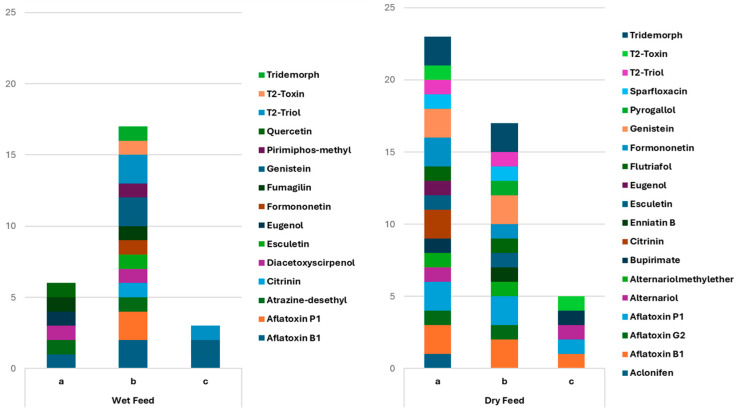
Sum of the compounds identified in wet (*n* = 3) and dry feeds (*n* = 3) using three different dSPE cleanup combinations (protocols a: 150 mg MgSO_4_ + 50 mg PSA + 50 mg C18; b: 300 mg MgSO_4_ + 50 mg PSA + 50 mg C18 and c: 50 mg Z-Sep+).

The MgSO_4_–PSA–C18 combinations surpassed Z-Sep+ for both feed types; however, the ideal quantity of MgSO_4_ was contingent upon the matrix characteristics. In high-moisture feeds, elevated MgSO_4_ levels increased dehydration and enabled the extraction of a larger spectrum of contaminants, whereas in dry feeds, reduced MgSO_4_ concentrations maintained greater chemical variety without significant loss of analytes. Figure 1 encapsulates these findings, indicating which protocol was optimal for each feed.

### 2.2. Suspect Screening

**Figure 2:**
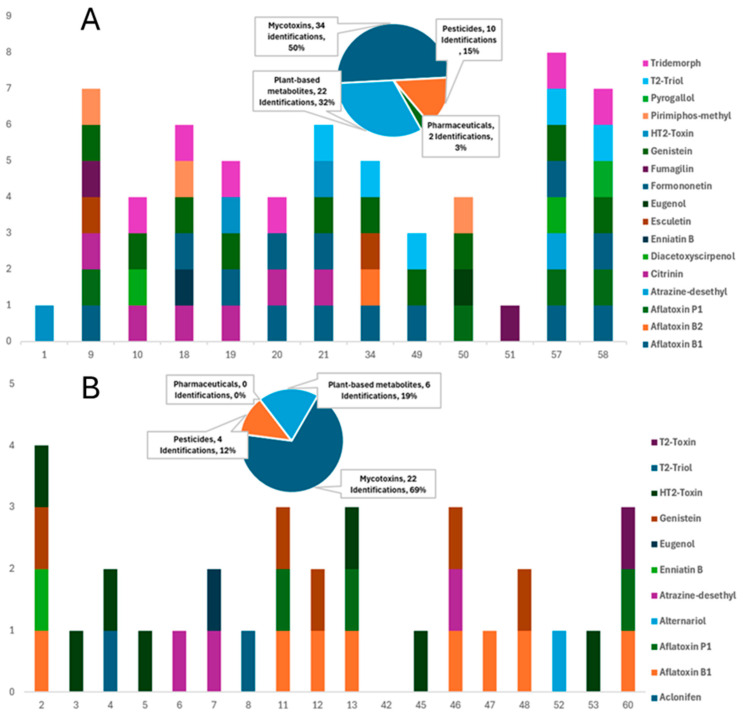
Comparative distribution of mycotoxins, pesticides, pharmaceuticals, and plant-based metabolites in: (**A**) dry dog feeds and (**B**) wet dog feeds (Values represent the number of different compounds detected per contaminant class in each group of feeds).

**Figure 3:**
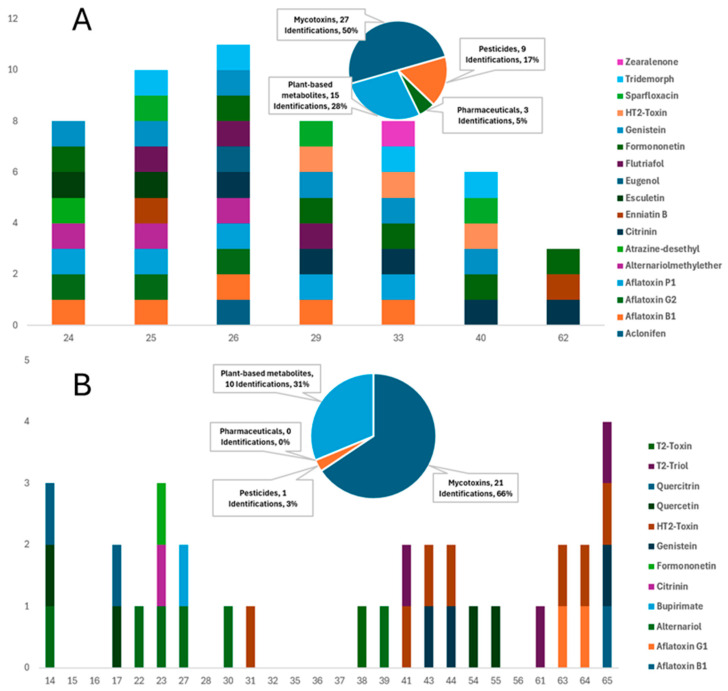
Comparative distribution of mycotoxins, pesticides, pharmaceuticals, and plant-based metabolites in: (**A**) dry cat feeds and (**B**) wet cat feeds (Values represent the number of different compounds detected per contaminant class in each group of feeds).

This study involved a thorough investigation of contaminants in commercially available dry and wet feeds for dogs and cats. Various compounds, including mycotoxins, pesticides, pharmaceuticals/veterinary drugs, and plant-based bioactive metabolites, have been tentatively identified in a large majority of the samples through the suspect screening strategy, demonstrating distinct qualitative detection patterns between dry and wet formulations (Table S3). Consequently, examining each category of contaminant individually, results underscore the potential health implications for pets, acknowledging that exposure and risk cannot be inferred in the absence of quantitative confirmation. A total of 29 compounds were tentatively identified in 65 samples, corresponding to 186 unique detections (Table 1). Dry formulations (DD for dry dog feed and DC for dry cat feed) accounted for 65.6% of these detections, while wet feeds (WD for wet dog feed and WC for wet cat feed) contained the remaining 34.4% (Figure 2 and Figure 3).

Mycotoxins have been recognized as the most prevalent contaminants, as they have been reported in 76.9% of all samples and 95% of dry formulations, indicating the sensitivity of cereal-based ingredients. Aflatoxin B1 was the most frequently detected mycotoxin, found in 26 samples, and was present in 71.4% of dry dog feed and 28% of dry cat feed. Additional mycotoxins, such as citrinin, HT-2 toxin, and Alternaria toxins, play an important role in the overall contamination profile. Pesticide residues were identified in 72% of dry feeds, while only 11% of wet feeds contained such residues, implying that these residues are associated with grain cultivation and storage practices. Plant-derived metabolites, such as flavonoids and phytoestrogens, including genistein and formononetin, were detected in 51% of the samples. The presence of botanical ingredients alongside mycotoxins and pesticides complicates risk attribution and indicates potential hormonal relevance, especially for cats, which exhibit limited metabolic tolerance for these compounds; however, in the absence of quantitative exposure data, no conclusions can be drawn regarding actual hormonal risks. At the same time, pharmaceutical residues were relatively few, yet significant, with fumagilin identified in three samples and sparfloxacin in one, overall accounting for 3% of all detections, in contrast to nearly 25% in dry feeds.

**Figure 4:**
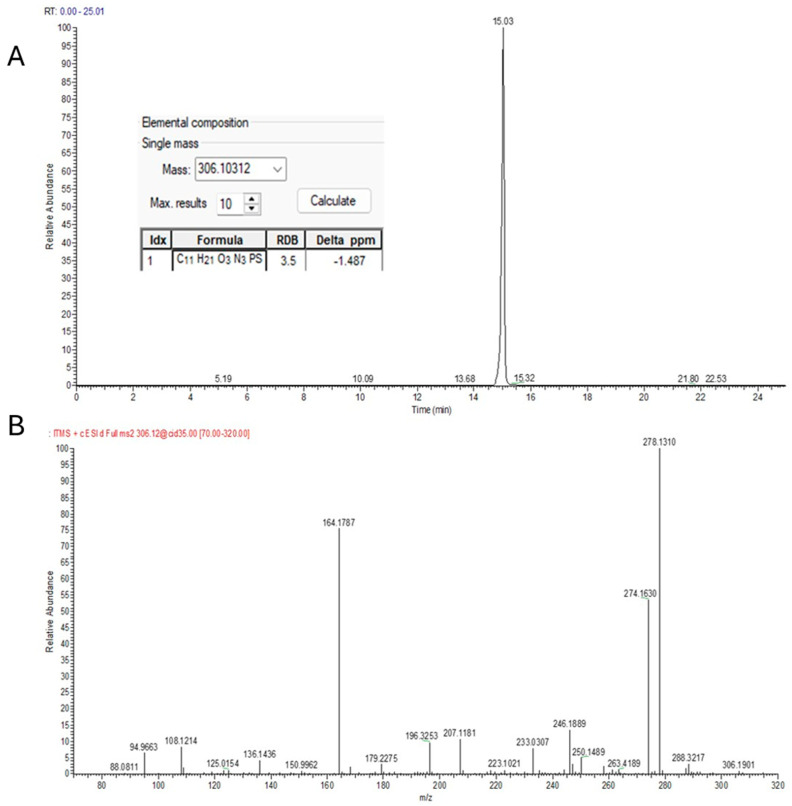

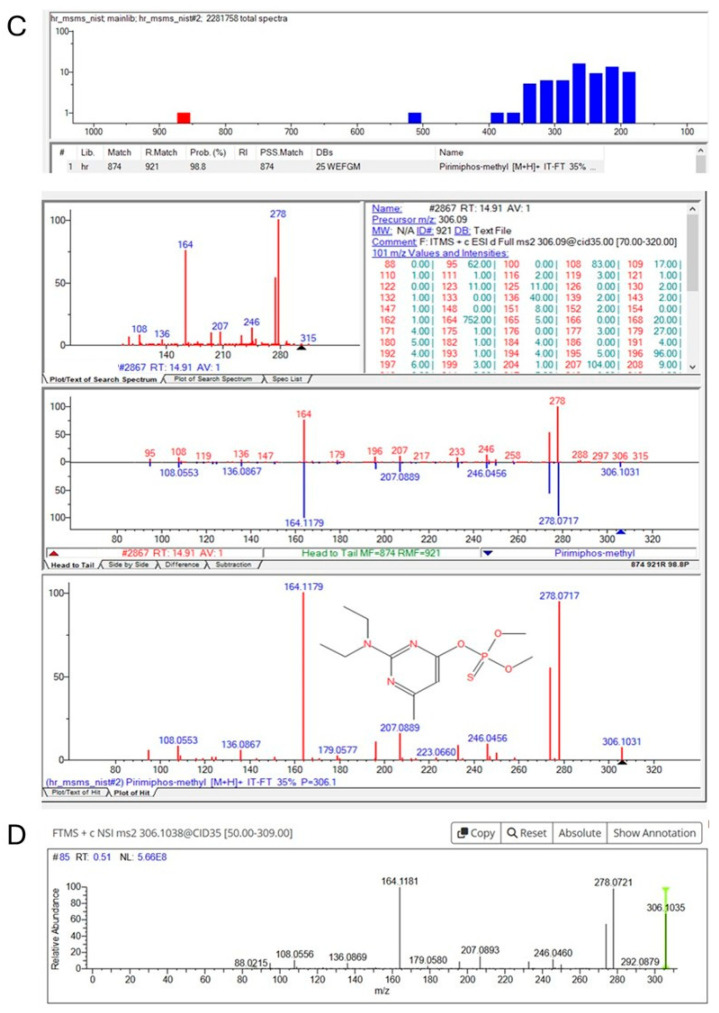
Identification of pirimiphos−methyl in pet feed using UHPLC−LTQ/Orbitrap HRMS. (**A**) Extracted ion chromatogram; (**B**) experimental MS^2^ spectrum; (**C**) library match with NIST; (**D**) confirmation through spectral comparison with mzCloud.

As shown in Figure 4 (pirimiphos-methyl), a typical identification through suspect screening was carried out via either mzCloud MS^2^ spectra comparison or NIST MS^2^ Library matching. In many cases, both spectra comparisons were achieved, resulting in a higher confidence level of the identifications.

#### 2.2.1. Mycotoxins

Mycotoxins such as aflatoxins (B1, B2, G1, G2, P1), T2 toxin, HT2 toxin, citrinin, and diacetoxyscirpenol were tentatively identified in 76.9% of the total samples. Particularly, dry dog feeds (DD) exhibited a large number of aflatoxins present in 9 out of 14 samples. Aflatoxins are extremely hazardous to both dogs and cats, principally impairing liver function and potentially inducing cancer with extended exposure [49]. Because of their diminished detoxifying capabilities, cats are particularly sensitive to mycotoxins, even in small quantities [49,50,51].

Additionally, the detection of trichothecene mycotoxins, such as T2 and HT2, along with aflatoxins in almost all dry cat feeds, indicated contamination during grain cultivation, as these substances are usually linked to cereal crops [52,53,54]. The resistance of these mycotoxins to processing and storage highlights the necessity for meticulous procurement of grains used in pet feed. Consistent screening and utilization of high-quality components may mitigate these dangers, as mycotoxins persist throughout food production and are not easily reduced [55]. Overall, aflatoxin B1 was the most frequent, detected in 22 samples (33.8%), followed by HT-2 toxin (30.8%) and aflatoxin P1 (20.0%). Co-occurrence was frequent, with six samples containing at least four different mycotoxins, mainly in DC and DD feeds, indicating the potential for cumulative toxic effects.

#### 2.2.2. Pesticides

Pesticide residues, such as aclonifen, atrazine-desethyl, tridemorph, and pirimiphos-methyl, were primarily detected in dry feed samples, with 72.0% of the dry products containing at least one pesticide. The results indicated that raw materials in dry feeds are possible sources of water contamination or may originate from the plant-based components of the feeds, given that they are widely utilized pesticides in agriculture [1,56]. Another important reason for the detection of these pesticides is their use during processing and storage of animal feed. Pesticides, such as pirimiphos-methyl, can be introduced and have been repeatedly detected in aquaculture feed samples, indicating their use in feed preservation or pest control during storage [1,57,58]. Pesticides were detected sporadically in 11% of the wet feed samples without generating any pattern.

Chronic use of low-dose pesticides in pets may lead to endocrine disruption, immunological suppression, poisoning, and various health complications [59,60]. In fact, pets consume almost the same feed daily, and even minimal pesticide concentrations might lead to a magnification of the risks, supporting prioritization for targeted monitoring with quantitative methods in materials utilized for pet feed production [56,61]. The most frequently detected pesticide was tridemorph (16.9%), followed by atrazine desethyl (7.7%) and pirimiphos-methyl (4.6%).

#### 2.2.3. Pharmaceuticals

Pharmaceuticals suspect lists, including veterinary drugs, revealed sparfloxacin (antibiotic), tentatively identified in three samples. The inclusion of antibiotics in pet diets may originate from raw materials produced from meat or other animal-derived products that undergo antibiotic treatment [62,63]. In addition, sparfloxacin, used for illness treatment, may foster antibiotic resistance in dogs with prolonged exposure, underscoring a public health hazard similar to that in human food systems [64,65]. Although pharmaceuticals accounted for only 3% of the total detections, their occurrence in dry feeds highlights a potential contamination route that warrants further attention.

#### 2.2.4. Plant-Based Bioactive Metabolites

Compounds such as esculetin, genistein, formononetin, quercetin, quercitrin, pyrogallol, and eugenol were detected in 51% of the feeds. Genistein and formononetin are phytoestrogens and plant-derived compounds that emulate estrogen in organisms. Although these compounds occur naturally, they may have cumulative effects that are toxicologically relevant due to endocrine activity reported in the literature for dogs and cats, particularly in neutered animals with possibly altered hormonal balances [66]. The presence of these compounds indicates that plant-based ingredients, such as legumes or grains, are utilized as supplements in pet meals and may unintentionally introduce these metabolites [67,68]. Obligate carnivores such as cats are poorly equipped to digest plant-based substances, and phytoestrogens may disrupt reproductive health and hormonal control [69]. This underscores the necessity of meticulously balancing the plant components in feline nutrition to prevent adverse health consequences. Compounds such as genistein (38.5%) and formononetin (21.5%) were the most frequently detected, followed by quercetin and esculetin (6.2% each), and smaller numbers of quercetin, eugenol, and pyrogallol. In some samples, this class of compounds was detected alongside pesticides and mycotoxins, complicating the source attribution.

### 2.3. Multivariate Analysis of Feed Composition and Contaminant Profiles

#### 2.3.1. Correlation Analysis Between Nutritional Parameters and Contaminants

**Figure 5:**
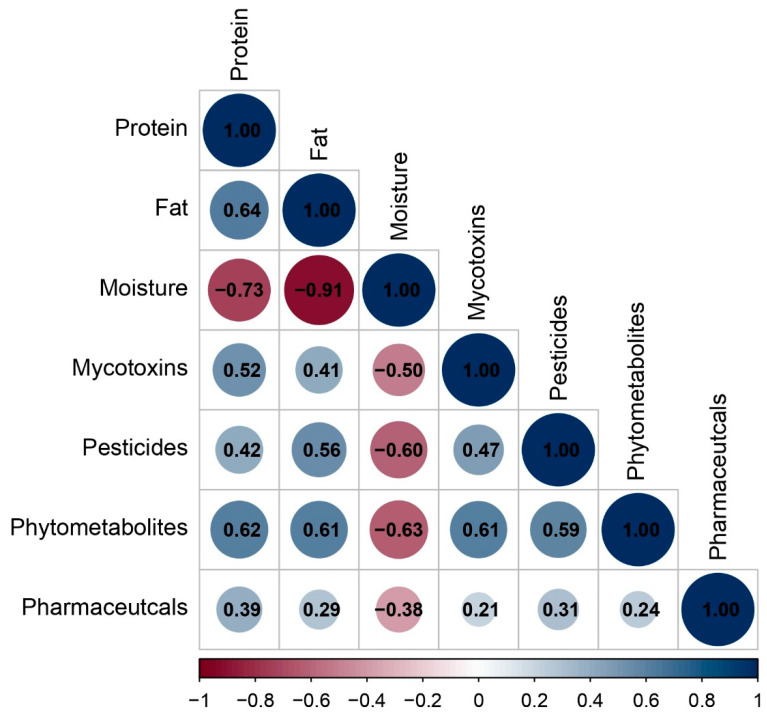
Spearman correlation heatmap (*p* < 0.05) showing trends between tentatively identified compounds and the nutritional components of the feeds; color intensity reflects the magnitude and sign of Spearman’s r.

Spearman correlation analysis (*p* < 0.05) revealed associations between contaminant categories and the nutritional composition of the feeds (Figure 5 and Table S4).

Pesticide residues (*r* = 0.56) and phytometabolites (*r* = 0.61) correlated positively with fat content and at the same time exhibited negative associations to moisture (*r* = −0.60 and −0.63, respectively). These observations align with lipophilicity-driven partitioning because many compounds of these categories have higher logK_ow_ values and therefore preferentially associate with lipid fractions.

Mycotoxins were positively correlated with phytometabolites (*r* = 0.61) and pesticides (*r* = 0.47). This correlation likely reflects shared agricultural origin, as both mycotoxins and phytometabolites originate from plant-based feed ingredients (cereals, legumes). The co-occurrence of multiple mycotoxins in feed has been widely reported [70] as has their presence in feed batches together with phytometabolites and pesticide-related compounds [71].

Correlations involving pharmaceuticals are generally weaker (*r* = 0.21–0.39). This is explained by considering the fact that pharmaceuticals span diverse properties and often arise episodically after targeted animal treatment, so their presence varies more with specific source ingredients than with bulk composition.

It is worth mentioning that commercial dry pet foods with low moisture are typically cereal-rich. On the other hand, high-moisture wet foods are mainly meat-based and cereal-poor. Thus, mycotoxins that are primarily cereal-borne tend to be higher in number where moisture is lower, simply because those products contain more cereal ingredients, leading to a negative correlation with moisture (*r* = −0.50).

Overall, the correlation results are consistent with: (i) compositional characteristics of the feed matrix, (ii) lipophilicity-driven partitioning of the tentatively identified compounds into the fat fraction, and (iii) shared agricultural origins of plant-based bioactives in feed ingredients. These findings indicate that the nutritional composition of pet feed significantly influences its detection diversity (number of tentatively identified compounds), which are indicative of both the sourcing of raw materials and the methods of processing.

#### 2.3.2. Principal Component Analysis of Composition–Contamination

**Figure 6:**
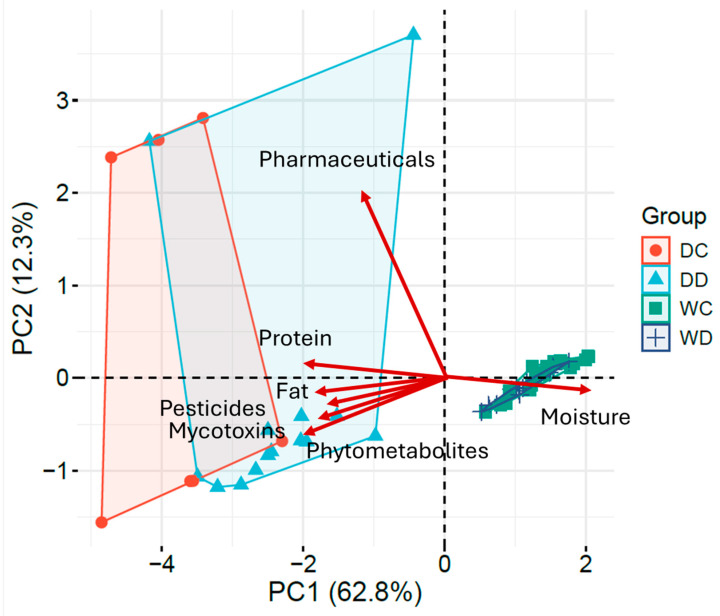
Principal component analysis and loading plots illustrating the contribution of nutritional parameters and contaminant categories: PC1 (62.8%) and PC2 (12.3%); scores are colored according to feed type (dry/wet, cat/dog), while red vectors represent the loadings of nutritional parameters and contaminant categories.

Principal Component Analysis (PCA) was employed to evaluate the relation of the feed composition to the occurrence of different contaminant classes (Table S5). The first three components explained 86.80% of the total variance, with PC1 describing 62.85%, PC2 12.35%, and PC3 11.61% of variability (Figure 6). In the biplot, each variable’s contribution is represented in the PC1-PC2 plane by the vector length, whereas the direction reflects the correlation of each variable with axes and other variables.

The first PC accounted for 62.8% of the total variability, contrasting moisture and exhibiting strong positive loading, with fat, protein and residue loads (negative loadings). Consequently, wet feed formulations (WD/WC) clustered at positive PC1, whereas dry ones clustered at negative PC1, which is indicative of a clear discrimination between them. The co-alignment of fat, protein, pesticides, mycotoxins, and phytometabolites indicates that cereal-based raw materials and fat content are major determinants of the contamination profile of dry formulations. Within this structure, samples characterized by high protein levels consistently clustered in the negative PC1 region and were associated with a higher number of mycotoxins tentatively identified, such as aflatoxin B1 and citrinin, together with pesticides, including pirimiphos-methyl. These were consistent with plant-derived ingredients in dry feeds.

PC2 (12.3%) was mostly driven by pharmaceutical variability and positively loaded, while the other variables showed near-zero loadings. This pattern emphasizes the rather episodic and largely independent-to-bulk-composition occurrence of these compounds.

The PCA biplot distinctly validated the separation between dry and wet formulations and aligned with correlation analysis: the nutritional composition of pet feed is the principal determinant of contaminant distribution except for pharmaceuticals that exhibit independent-composition behavior. The categorization of samples based on feed type and contaminant profile demonstrates that the sourcing and processing procedures of raw materials are essential for influencing contamination patterns.

### 2.4. Comparison Between Feed Types

#### 2.4.1. Dry Feeds

Dry feeds contain a broad range of pollutants, including pesticides, mycotoxins, pharmaceuticals, and plant-based bioactive metabolites (Figure 2 and Figure 3). In order to elaborate, mycotoxins were the most commonly identified group of compounds in dry samples (95%), indicating the presence of contaminated grains [52,53,54]. These compounds are associated with hepatotoxicity, immunosuppression, and possible carcinogenic consequences in dogs, demanding rigorous quality control measures [35]. In contrast, dry cat feeds were characterized by the presence of trichothecene mycotoxins and phytoestrogens, suggesting contamination from both grains and plant-derived components [49,50,51]. These findings are alarming because of the heightened sensitivity of cats to feed toxins. The detection of the HT-2 toxin indicates the necessity for rigorous regulation of cereal-based ingredients, as both substances are harmful to cats.

In addition, the detection of pharmaceuticals in 23.8% of dry feeds is a noteworthy outcome of suspect screening. This finding requires attention in future analytical campaigns, particularly those focused on antibiotics such as sparfloxacin, which may have significant implications for animals that consistently consume dry feeds. These results suggest potential contamination routes from animal-derived substances, where raw materials may originate from animals subjected to antibiotic treatment [1,57,58,62,63]. Furthermore, the frequent presence of pesticide residues in all dry feeds (71.5%) verified their wide use in grain cultivation and storage preservation. Overall, DD exhibited 68 detections across 17 compounds, whereas DC contained 54 detections across 17 compounds. DD was dominated by aflatoxin B1, citrinin, and HT-2 toxin, whereas DC showed a higher number of co-occurring mycotoxins, with 4 samples containing five or more contaminants. Thus, DD carried a more complex contamination profile, while DC displayed a greater multiresidue complexity.

#### 2.4.2. Wet Feeds

In contrast to dry feeds, wet feeds exhibited lower contamination loads, presenting lower detection rates across all categories of analytes (Figure 2 and Figure 3). Suspect screening revealed the presence of mycotoxins, pesticides, and phytometabolites in all samples of this group. Mycotoxins were present in 30 of the 44 samples, including members of different mycotoxin classes, such as aflatoxins, Alternaria, and trichothecene mycotoxins. The low percentage of pesticide occurrence verified the increased need for these compounds in the storage preservation of dry feeds, as only 11% of wet samples contained pesticide residues. Considering the nutritional requirements of cats, contamination with phytoestrogens (such as formononetin and genistein) is particularly concerning, as these substances can disrupt hormonal equilibrium and potentially hinder normal biological processes [69]. This contamination pattern underscores the necessity of regulatory supervision in the procurement and processing of raw materials for wet pet feed manufacturing to alleviate these risks. Among the wet products, WD contained 32 detections across 11 compounds, dominated by aflatoxin B1 (8 detections) and HT-2-Toxin (7), with sporadic findings of sparfloxacin and fumagilin. WC contained 32 detections across 12 compounds, but uniquely featured Alternaria toxins (alternariol in 6 samples) and phenolic metabolites, such as quercitrin. Thus, WD showed a higher prevalence of regulated contaminants, while WC was characterized by emerging toxins and natural metabolites.

### 2.5. Practical Implications and Limitations

Τhe combined presence of mycotoxins, pesticides, veterinary drugs and plant-based bioactives suggests that companion animals, particularly cats with limited metabolic capacity to detoxify xenobiotics, may be exposed to complex mixtures of contaminants with possible additive or synergistic effects; nonetheless, the absence of quantitative concentration data does not allow any conclusions about the actual magnitude of exposure or risks, given the current absence of pet-specific regulatory limits for most emerging contaminants in pet feeds. These findings point to a critical regulatory gap and advocate for the integration of advanced non-target/suspect strategies using HRMS, complemented by confirmatory targeted approaches, into routine quality controls. Establishing monitoring frameworks specifically for pet feeds would provide an essential basis for improving feed safety management and, indirectly, public health protection in households where pets are considered family members.

This study has some limitations that should be acknowledged when interpreting the results. The qualitative nature of suspect screening prevents precise risk assessment studies in the absence of reference standards and robust concentration data. In this context, the findings should not be interpreted as evidence of regulatory non-compliance or actual toxicity. Future work could therefore include targeted quantitation for priority contaminants tentatively identified here, since compound-specific LODs/LOQs, recoveries and precision parameters were not determined in this study. Instead, the proposed analytical approach should be regarded as comprehensive screening and the first step in identifying contaminants of concern in pet feeds for future targeted quantitation and risk assessment. The sample set (despite the sufficient number: 65 samples) was limited to a single sampling campaign, which may restrict the generalizability of the findings and does not capture seasonal or batch-to-batch variability. Overall, the dataset should be regarded as a regional case study based on widely marketed products, illustrating the applicability of LC-HRMS suspect screening to commercial pet feeds and providing a basis for designing broader, multi-regional and longitudinal monitoring efforts. Furthermore, identifications at Schymanski Level 2 inherently carry some degree of structural uncertainty and should be interpreted as qualitative evidence of presence with stated confidence, requiring confirmation with authentic standards where feasible. Therefore, the findings should not be interpreted as definitive confirmation for all compounds nor as evidence of regulatory non-compliance. Where available, authentic standards were analysed and used to confirm selected annotations (Level 1).

Despite these limitations, the present study clearly demonstrated the value of HRMS-based suspect screening as an early-warning tool, providing mechanistic insight into contamination sources and co-occurrence patterns. In particular, this early-warning capacity supports evidence-based prioritization of contaminants or their mixtures for feed safety management, particularly in matrices and contaminant classes where routine targeted monitoring remains limited.

## 3. Conclusions

In this study, a comprehensive LC-HRMS workflow was employed for the suspect screening of 65 commercial cat and dog feeds. To the best of our knowledge, it is the first systematic comparison of contamination profiles between dry and wet formulations in a single multi-class analytical run. The results revealed clearly diverse contamination patterns driven by feed composition (moisture, fat and protein) and raw material sourcing, with dry formulations exhibiting an approximately four-fold higher number of identified compounds (122 detections) compared to wet feeds (64 detections). Mycotoxins and pesticides were prevalent in dry formulations, consistent with the higher inclusion of cereal-based ingredients and associated agricultural practices. Also, plant-based bioactive metabolites were identified in both dry and wet feeds, indicating an additional, unregulated source of bioactives in pet nutrition. The frequent co-occurrence of multiple mycotoxins further underscores the complexity of actual exposure scenarios that are not adequately captured by single-compound regulatory approaches. Overall, HRMS-based suspect screening offers an effective early-warning and prioritization approach for complex feed matrices. Quantitative confirmation and mixture-oriented assessment would be required to translate these findings into exposure- and risk-based conclusions.

## 4. Materials and Methods

### 4.1. Sampling Campaign

A total of 65 commercial pet feed samples were collected including 33 cat feeds (7 dry and 26 wet) and 32 dog feeds (14 dry and 18 wet). One unit of each distinct commercial product that fulfilled the inclusion criteria was purchased from large supermarket chains and veterinary stores in the region of Ioannina (NW Greece). The sampling campaign (May 2024) followed the official protocols outlined in Commission Regulation (EC) No 401/2006 and Commission Directive 2002/63/EC where applicable. The selected feeds belonged to major brands that are widely marketed across Greece and, in many cases, other European countries. Thus, although the purchases were made in a single region, the products themselves are representative of commonly used commercial pet feeds in Greek and European markets. The sampling was designed as a market-basket snapshot of the products available locally during the sampling period. The different numbers of wet and dry feeds per species thus reflect the actual distribution and diversity of products available in the Greek market at the time of sampling, rather than a pre-defined balanced experimental design. The samples included feed formulations intended for feline and canine nutrition. Selection criteria considered feed type, ingredient composition, and estimated frequency of consumption associated with each product. According to the label declarations, the dry formulations corresponded to typical cereal-based kibbles combining cereals with meat and animal derivatives, vegetable protein sources, fats and minerals, whereas the wet formulations predominantly contained meat and animal derivatives and/or fish and fish derivatives, with cereals present in some products. All samples were categorized into two primary classifications, animal species (cat and dog), followed by a subsequent categorization of protein, fat and moisture content (Table S1).

### 4.2. Materials and Reagents

All solvents used in the analysis were of LC-MS grade and included water, methanol, and acetonitrile (Fisher Scientific, Waltham, MA, UK). Formic acid (FA, purity 98–100%) and ammonium formate (NH_4_FA, ≥98%) were obtained from Merck (Darmstadt, Germany). QuEChERS extraction salts were also purchased from Merck and included anhydrous magnesium sulfate (MgSO_4_), C18 sorbent (LiChroprep RP-18, 40–64 μm), and trisodium citrate dihydrate (C_6_H_5_Na_3_O_7_·H_2_O). Additional reagents such as sodium acetate (NaOAc) and sodium chloride (NaCl) were acquired from Riedel-de Haën (Hannover, Germany), while sodium citrate dibasic sesquihydrate (C_6_H_6_Na_2_O_7_·1.5H_2_O) was purchased from Sigma-Aldrich (Steinheim, Germany). Primary secondary amine (PSA; 40 μm) was provided by Agilent Technologies (Waldbronn, Germany). Syringe membrane filters made of polytetrafluoroethylene (PTFE, 0.22 μm) were purchased from Millipore (Cork, Ireland). All sample preparation steps were conducted using 50 mL and 15 mL polypropylene centrifuge tubes.

### 4.3. Sample Preparation

The buffered QuEChERS extraction method was chosen for suspect screening. The experimental procedure followed the standardized multiresidue approach commonly employed in previous studies [1,72]. Prior to extraction, all dry feed samples (kibbles) were ground using a laboratory mill to obtain a fine, homogeneous powder, while wet feed samples were thoroughly mixed with a stainless-steel spatula to form a uniform paste. Five grams (5 g) of homogenized sample was weighed into a 50 mL polypropylene centrifuge tube, followed by the addition of 10 mL of LC-MS grade water and 10 mL of LC-MS grade acetonitrile. The mixture was agitated on a horizontal shaker for 15 min. Subsequently, extraction salts were added and the tube was manually shaken for 1 min, followed by vortexing for an additional minute to minimize coagulation during MgSO_4_ hydration. The samples were then centrifuged at 4000 rpm for 5 min. An aliquot of 1 mL of the supernatant was transferred into a clean 15 mL tube containing cleanup salts. After vortexing for 1 min and centrifugation for 5 min (4000 rpm), 1 mL of the final extract was filtered through a 0.22 μm PTFE syringe filter and transferred to vials for analysis. The final extracts were analyzed using an LC-HRMS system described in Section 4.4. 

### 4.4. LC-LTQ/Orbitrap MS Analysis

A UHPLC LTQ/Orbitrap MS system was used to analyze all samples. Chromatographic separation was performed using an Accela LC system (Thermo Fisher Scientific, Bremen, Germany), which comprised an Accela AS autosampler (model 2.1.1) and Accela quaternary gradient LC pump. The liquid chromatography system was integrated with an LTQ-FT Orbitrap XL 2.5.5 SP1 mass spectrometer (Thermo Fisher Scientific Inc. GmbH, Bremen, Germany). The Linear Ion Trap (LTQ) component of the hybrid mass spectrometry system was equipped with an Εlectrospray Ιonization (ESI) probe that functioned in both positive and negative ionization modes. A reversed-phase Hypersil GOLD C18 analytical column (100 mm × 2.1 mm, 1.9 μm) from Thermo (Bremen, Germany) was employed for the separation of chemicals in the suspect screening of organic compounds of interest. The mobile phase consisted of phase A (0.1% formic acid in water) and phase B (0.1% formic acid in methanol), using a gradient elution technique that commenced at 98% mobile phase A (Table S2). The flow rate was established at 0.4 mL per minute. The overall run time of the program was 25 min. The injection volume was 10 μL, with tray and oven temperatures of 15 and 40 °C, respectively.

Full-scan MS spectra were obtained in the Orbitrap mass analyzer (Thermo Fisher Scientific, Bremen, Germany) (mass range m/z 100–1500) in positive ionization mode. Data-dependent MS/MS was triggered on the top-six most intense ions in the Linear Ion Trap. A normalized collision energy (NCE) of 35% was employed in all instances to obtain detailed fragmentation patterns. All the identified chemicals were characterized according to their molecular ion formation ([M+H]^+^, [M+Na]^+^). Source parameters (spray voltage, capillary temperature, sheath/aux gas) and Orbitrap settings (resolution, AGC target, maximum injection time) are reported in Table S2. Their distinctive fragments were analyzed against current mass spectrum databases (mzCloud^TM^, NIST, MassBank, Pubchem) or referenced literature when database access was unfeasible. Instrument control and mass spectral analysis were conducted using the Xcalibur v.2.2 software (Thermo Electron, San Jose, CA, USA).

### 4.5. Data Processing and Compound Identification

LC-HRMS data were processed by Compound Discoverer 3.3™ software (Thermo Fisher Scientific, Waltham, MA, USA, demo version) using a custom workflow (Figure S1). A categorical factor was first used to distinguish between wet and dry feed formulations. Three types of samples were assigned, comprising “quality control” for the matrix-matched samples, “sample” for the unknown ones and “blank” for the solvent samples. A chromatographic signal-to-noise (S/N) threshold of 1.5 was applied in order to account for as many identifications as possible. The protonated forms were included. The parameters of the connecting nodes were mostly kept in the default mode or adjusted as previously described [40], except for specific changes necessary to this study; tentative identifications used the “Assign Compound Annotations” node with 10 ppm mass tolerance for precursor ions and 300 ppm for the fragment matching obtained in the LTQ low-resolution mass analyzer. A minimum intensity threshold of 1 × 10^4^ was applied. The ChemSpider was used for parent mass and elemental composition searches as an additional source of identification. The “Search Mass Lists” node was utilized with many loaded mass lists, including a previously used [39] home-made mass list with suspect pesticides, new ones oriented to mycotoxins and pharmaceutical, including veterinary drugs compiled from literature data on feed studies, and the inherent EFS HRAM Compound Database. MS/MS fragmentation patterns were manually compared with those obtained by standards, if available, from either existing data in online and commercially available databases (mzCloud, NIST MS/MS library) or from reported data in the literature to tentatively confirm the suspect compounds’ identifications. Experimental MS/MS product ions and retention times supporting each annotation are provided in Table S6. According to the Schymanski rules [73], a level of identification was assigned to each suspect compound. Level 1 identifications were assigned only when confirmed with authentic reference standards analysed under the same conditions. Level 2 annotations were reported as probable structures supported by accurate mass and MS/MS evidence (and the respective experimental product ions and retention times are provided in Table S6). Each candidate was checked manually for peak shape reliability and LC-MS amenability.

In line with the qualitative, screening design of the study, no compound-specific limits of detection or quantification (LOD/LOQ), recoveries or precision (%RSD) were calculated, and the proposed methodology was therefore not validated for targeted quantitative purposes. The QuEChERS extraction and LC-HRMS conditions were, however, based on a previously validated multi-residue protocol for aquaculture fish feeds [1], where method detection limits in the range 0.2–15 ng g^−1^ and quantification limits in the range 0.5–50 ng g^−1^ were achieved for representative pesticides and pharmaceuticals, with recoveries generally within 70–120% and precision (RSD) below 20%.

### 4.6. Statistics

Exploratory data analysis was performed on the tentatively identified compounds based on the HRMS results. For each contaminant class (mycotoxins, pesticides, veterinary drugs, plant-based bioactive metabolites), the number of different compounds detected per sample was recorded as a measure of chemical diversity. All statistical analyses were conducted employing the SRPlot bioinformatics platform [74]. Spearman’s rank correlation was employed to assess relationships between nutritional composition (protein, moisture and fat content expressed as % *w*/*w*) variables and contaminant class diversity (expressed as detection counts per sample), applying a significance threshold of *p* < 0.05. Principal Component Analysis (PCA) was conducted on standardised (mean-scaled and scaled to unit variance) data to assess the variance structure of the dataset. The number of retained components and the explained variance followed default SRPlot parameters for eigenvalue calculation and biplot creation.

## Figures and Tables

**Table 1:** Summary of compounds tentatively identified in commercial cat and dog feeds by LC-LTQ/Orbitrap HRMS suspect screening, including the total number of identifications, distribution between wet and dry feeds, detection frequency in the dataset (*n* = 65 samples), Schymanski confidence level and reference sources used for identification such as mzcloud, (https://www.mzcloud.org/, accessed on 29 December 2025), MassBank (https://massbank.eu/MassBank/, accessed on 29 December 2025), Pubchem (https://pubchem.ncbi.nlm.nih.gov/, accessed on 29 December 2025), NIST MS Search (Version 3.0 build 18 April 2023) and resarch papers [48].

Compound	Total Identifications	Wet Feed Identifications	Dry Feed Identifications	Detection Frequency % ^1^	Schymanski Levels ^2^	Reference
Aclonifen	2	1	1	3.1%	2	Pubchem, MassBank, NIST
Aflatoxin B1	22	9	13	33.8%	2	mzCloud, MassBank, NIST
Aflatoxin B2	1	0	1	1.5%	2	mzCloud, MassBank, NIST
Aflatoxin G1	2	2	0	3.1%	2	mzCloud, MassBank, NIST
Aflatoxin G2	3	0	3	4.6%	2	mzCloud, MassBank, NIST
Aflatoxin P1	13	3	10	20.0%	2	Pubchem, [48]
Alternariol	7	7	0	10.8%	1	Standard
Alternariolmethylether	3	0	3	4.6%	2	mzCloud, MassBank, NIST
Atrazine-desethyl	5	3	2	7.7%	2	mzCloud, MassBank, NIST
Bupirimate	1	1	0	1.5%	1	Standard
Citrinin	13	1	12	20.0%	1	Standard
Diacetoxyscirpenol	2	0	2	3.1%	2	mzCloud, MassBank, NIST
Enniatin B	4	1	3	6.2%	2	mzCloud, MassBank, NIST
Esculetin	4	0	4	6.2%	2	mzCloud, MassBank, NIST
Eugenol	3	1	2	4.6%	2	mzCloud, MassBank, NIST
Flutriafol	3	0	3	4.6%	1	Standard
Formononetin	14	1	13	21.5%	2	mzCloud, MassBank, NIST
Fumagilin	2	0	2	3.1%	2	NIST, [48]
Genistein	25	8	17	38.5%	2	mzCloud, MassBank, NIST
HT2-Toxin	20	14	6	30.8%	1	Standard
Pirimiphos-methyl	3	0	3	4.6%	1	Standard
Pyrogallol	1	0	1	1.5%	2	mzCloud, MassBank, NIST
Quercetin	4	4	0	6.2%	2	mzCloud, MassBank, NIST
Quercitrin	2	2	0	3.1%	2	mzCloud, MassBank, NIST
Sparfloxacin	3	0	3	4.6%	2	mzCloud, NIST
T2-Toxin	10	4	6	15.4%	1	Standard
T2-Triol	2	2	0	3.1%	2	[48]
Tridemorph	11	0	11	16.9%	1	Standard
Zearalenone	1	0	1	1.5%	1	Standard

^1^ Detection frequency (%) = (number of samples in which a compound was detected/65 total samples) × 100. ^2^ Schymanski levels are assigned according to Schymanski rules and reflect the confidence in identification (Level 1: confirmed structure with reference standard; Level 2: probable structure based on diagnostic evidence).

## Data Availability

The original contributions presented in this study are included in the article/Supplementary Material. Further inquiries can be directed to the corresponding authors.

## Supplementary Materials

The following supporting information can be downloaded at: https://www.mdpi.com/article/10.3390/toxins18010022/s1, Figure S1: Custom workflow of compound discoverer 3.3 for the suspect screening of feed samples; Table S1: Nutritional composition (protein, fat, and moisture) of commercial dog and cat feeds, separated by dry and wet formulations.; Table S2: UHPLC gradient elution program and MS parameters of LTQ-Orbitrap HRMS applied for the separation of organic contaminants in pet feed; Table S3: Raw detections of contaminants per sample (Data derived from the 65 analyzed feed samples (DD = dry dog feed, DC = dry cat feed, WD = wet dog feed, WC = wet cat feed). Presence = 1, Absence = 0.); Table S4: Correlation coefficients (r-values) between nutritional parameters and contaminant categories; Table S5: PCA loadings of nutritional parameters and contaminant categories. Table S6. Analysis of suspect screening by UHPLC-LTQ-ORBITRAP-MS.
